# Global research trends and hotspots in patellofemoral pain syndrome from 2000 to 2023: a bibliometric and visualization study

**DOI:** 10.3389/fmed.2024.1370258

**Published:** 2024-03-19

**Authors:** Jie Xu, Zijuan Cai, Meng Chen, Xin Wang, Xiaobing Luo, Yanjie Wang

**Affiliations:** ^1^Department of Sports Medicine, Sichuan Provincial Orthopedics Hospital, Chengdu, China; ^2^College of Physical Education and Health, Geely University of China, Chengdu, China; ^3^Department of Emergency Medicine, Nanchong Hospital of Traditional Chinese Medicine, Nanchong, China; ^4^Health Science Center, Peking University, Beijing, China

**Keywords:** patellofemoral pain syndrome, bibliometrics, PFPS, rehabilitation, CiteSpace

## Abstract

**Background:**

Patellofemoral pain syndrome (PFPS) is a prevalent condition in sports medicine, and as sports competitions become more popular, the incidence of sports injuries is on the rise. Despite the increasing research on PFPS, there remains a lack of bibliometric analyses on this topic. The aim of this study was to identify the research hotspots and trends in the field of PFPS by reviewing 23 years of literature in this field.

**Methods:**

By analyzing the literature on PFPS research from 2000 to 2023 in the core dataset of the Web of Science database and utilizing bibliometric tools like CiteSpace 6.1, VOSviewer 1.6.18, R-bibliometrix 4.6.1, Pajek 5.16, and Scimago Graphica 1.0.26, our aim was to gain insights into the current status and key areas of PFPS research. The study examined various aspects including the number of publications, countries, institutions, journals, authors, collaborative networks, keywords, and more. Through the visualization of relevant data, we also attempted to forecast future trends in the field.

**Results:**

There were 2,444 publications were included in this visualization study, published in 322 journals by 1,247 authors from 818 institutions in 67 countries. The Journal of Orthopaedic and Sports Physical Therapy had the highest number of publications, with the USA leading in article count. La Trobe University contributed the most articles, while Rathleff MS and Barton CJ emerged as the most prolific authors. Hip and knee strength and core strength, lower extremity kinematics and biomechanics, females (runners), muscle activation, risk factors, gait retraining, clinical practice guidelines, and rehabilitation were research hotspot keywords.

**Conclusion:**

Current research suggests that there is still significant potential for the development of PFPS research. Key areas of focus include the clinical effectiveness of combined hip and knee strengthening to address PFPS, characterization of lower limb kinematics and biomechanics, gait retraining, risk factors, and clinical practice guidelines. Future research could explore the effectiveness of innovative exercise therapies such as blood flow restricting training, gait retraining, and neuromuscular control training for PFPS improvement. Further investigation into gait retraining for runners, particularly females, and clinical efficacy study of a novel PRP formulation for the treatment of PFPS.

## 1 Introduction

Patellofemoral pain syndrome (PFPS) is a prevalent condition affecting adolescents and active adults, characterized by diffuse knee pain exacerbated by weight bearing and knee flexion. The squat test is a reliable physical examination method for diagnosing PFPS, which is considered one of the most common knee ailments (1). Young female patients without structural alterations, like an elevated Q-angle or severe cartilage injury, are often affected by PFPS, a potential cause of anterior knee discomfort (2). The prevalence of PFPS in adolescents is between 20 and 40%, with around 1 in 10 male recruits experiencing the condition (3). A study on a mixed-gender recruitment population found that the prevalence of the condition in females was about 2.23 times higher than in males (4). The annual prevalence rate for the general adult population was reported to be 22.7%, with rates of 29.2% for women and 15.5% for men (5). Another study focusing on male cyclists revealed that 35.7% experienced pain symptoms yearly, with 6.4% enduring symptoms for over 30 days (6). For instance, in the UK, an estimated $21,300 million is required annually for disease treatment (7). In recent years, there has been increasing evidence that early treatment without timely and effective treatment may eventually lead to irreversible patellofemoral arthritis (8, 9). In the case of patellofemoral arthritis, surgical treatment, such as joint replacement, is required, increasing the difficulty of cure, prolonging the treatment period, and causing serious financial losses (10). Most importantly, the quality of life and motor function of patients are seriously affected (11). Given its high incidence and prevalence, poor long-term prognosis, and significant disability levels, PFPS stands as a critical research priority. Despite notable advancements in treatment methods and their effectiveness over the years, ongoing debates persist. The introduction of new theories and technologies has presented both opportunities and challenges in treatment, underscoring the importance of exploring current hotspots and research trends in PFPS. CiteSpace and VOSviewer software are capable of generating co-citation networks by analyzing reference citations, thereby illustrating the structure of a specific research field. Through the examination of countries, authors, journals, institutions, and keywords within a field, these tools can provide insights into the development trends and research hotspots. Bibliometrics has gained popularity across diverse fields in recent years due to its straightforward, intuitive, and objective nature (12). While numerous reviews have been conducted on the treatment of PFPS, there remains a gap in systematic bibliometric analyses of PFPS. This study seeks to review articles published on PFPS from the past 23 years to identify research hotspots and trends in the field. The goal is to offer valuable insights and references for future studies.

## 2 Methods

### 2.1 Data sources and retrieval strategies

**Figure 1:**
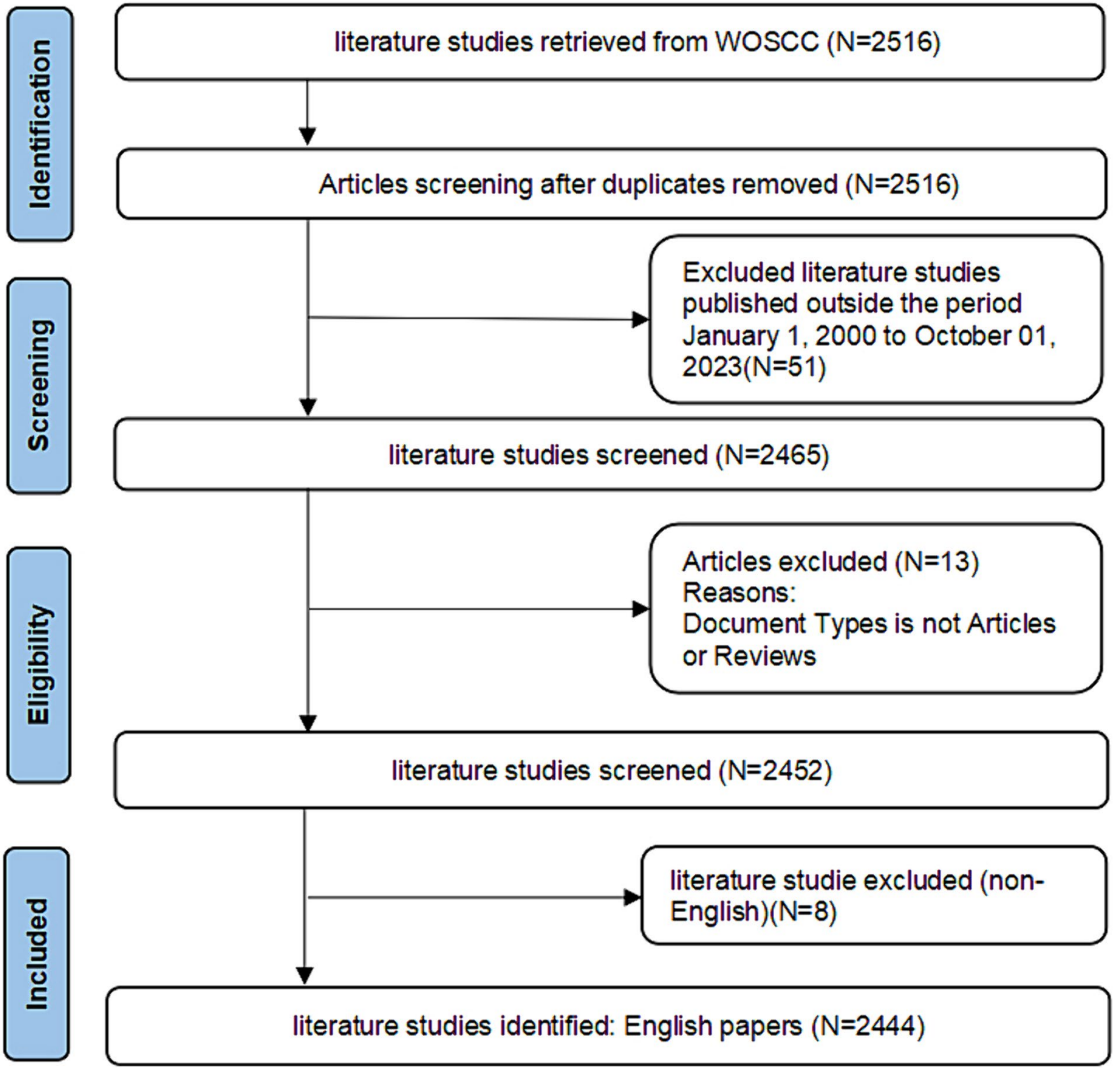
Workflow diagram.

In this study, compliance with bibliometric analyses was ensured by aligning the selection criteria and operational procedures with those outlined by Prof. Chen (13), the founder of CiteSpace software, and other highly impactful bibliometric literature (14–16). The Web of Science database core dataset was searched with the search terms “TS = (Patellofemoral Pain OR Chondromalacia patella OR Patella-femoral pain syndrome OR patellofemoral pain syndrome OR Anterior Knee Pain Syndrome).” The research in this field was first documented in 1979, however, only 51 articles were published between 1979 and 2000. This limited number of publications, with some years having no articles published, resulted in poor visualization effects. To address this, the search time range was set from 1 January 2000 to 1 October 2023. Literature type was restricted to “Article” and “Review” to better reflect the development timeline of the discipline. Given that most literature in the Web of Science database is in English, and high-impact bibliometric studies include only English language literature, so this study also includes only English language literature. The remaining selection criteria aligned with established high-impact bibliometric research standards. Literature was selected for this study based on the following inclusion and exclusion criteria: (i) the literature was published between 1 January 2000 and 1 October 2023; (ii) the type of literature was Article and Review; (iii) the language of the literature was English; (iv) literature such as conference abstracts, proofreading notices, news, conference papers, and retractions notices were excluded. Ethical approval was not required as the data in the articles did not contain any personal information of the patients, and 2,444 publications remained after removal. Figure 1 displays the process flowchart in question.

### 2.2 Literature selection

The literature was independently reviewed by two evaluators. The title and abstract of the literature were used for the initial screening, followed by a second screening based on inclusion and exclusion criteria. In cases of disagreement during screening, a third assessor reviewed the full article and made the final decision on acceptance or rejection. To withdraw a document, navigate to the “Plain Text” folder where the document is stored in notepad form. Search for the document by its title and delete it to withdraw the document.

### 2.3 Data analysis

This bibliometric analysis study utilized a range of tools including CiteSpace version 6.1.R6, VOSviewer version 1.6.18, R-Studio-based R-bibliometrix version 4.6.1, Pajek version 5.16, and Scimago Graphica version 1.0.26. CiteSpace was employed for visualization analysis, covering aspects such as country and institution distribution, journal citations, reference analysis, keyword analysis, and citation bursts (17). Detailed parameters can be found in Supplementary Table 1. VOSviewer primarily examines co-occurrence relationships within literature. By analyzing co-occurrence frequencies, researchers can identify common themes and concepts, and generate co-occurrence network graphs for visualization (18). Detailed parameters can be found in Supplementary Table 2. Cluster analysis can be utilized to identify research hotspots or sub-domains within the network of academic papers (19). Tools such as R-Studio based R-bibliometrix version 4.6.1, Pajek version 5.16, and Scimago Graphica version 1.0.26 are effective for multi-modal and multi-dimensional geo-visualization. In this study, it was used to highlight the inter-cooperative network relationships between different countries or regions. By utilizing overlap analysis with tools such as R-bibliometrix, VOSviewer, and CiteSpace, researchers can pinpoint research frontiers with the potential for significant advances in the near future. This approach allows for a comprehensive examination of the domain, enabling researchers to quickly understand the current body of knowledge and emerging trends in the field, ultimately improving their research outcomes and strategies.

## 3 Results

### 3.1 Trends in the quantity of published articles

**Figure 2:**
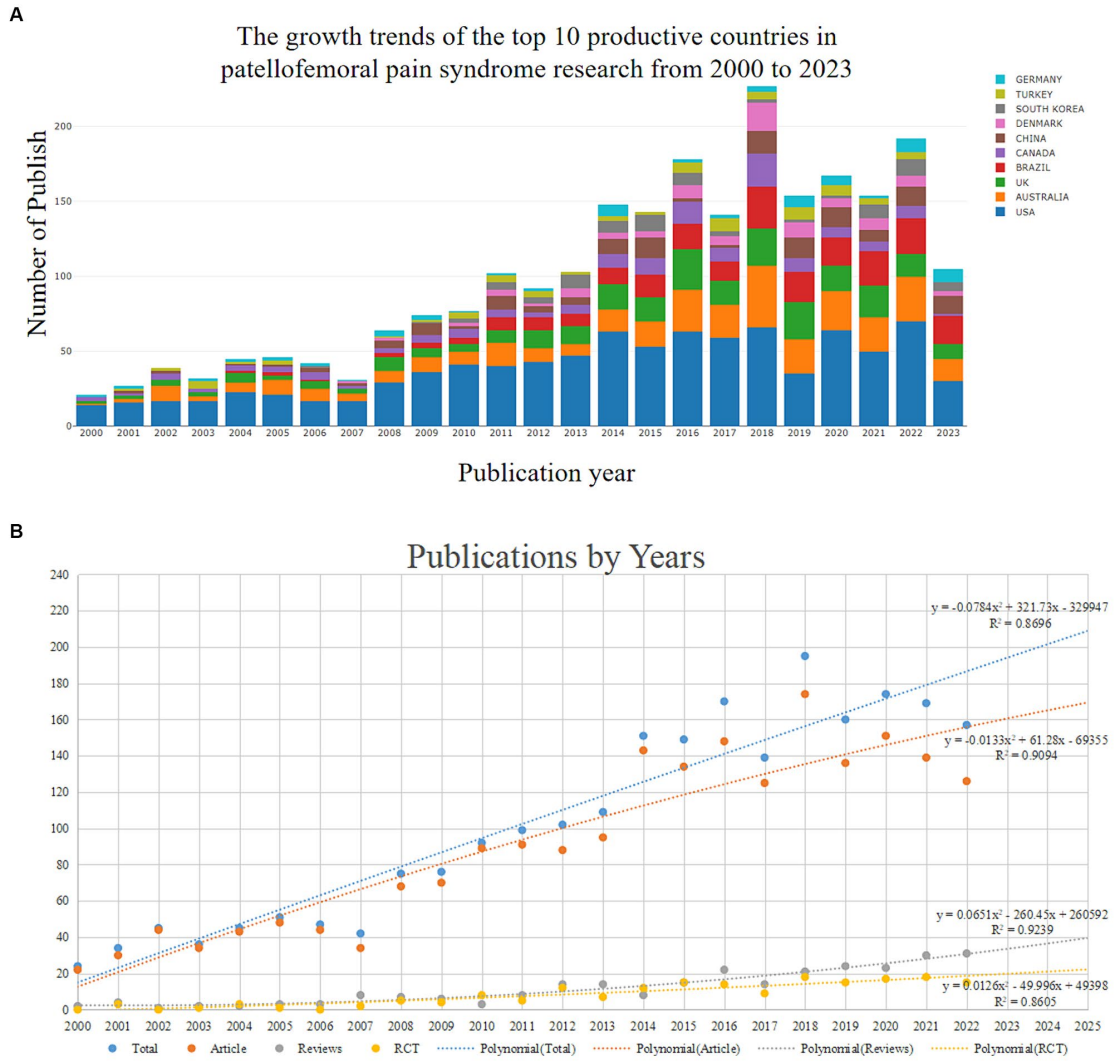
**(A)** Bibliometric analysis of the WoS core database output. **(B)** Publication trends in the PFPS field and the corresponding polynomial fit curves.

Figure 2A displays the yearly publication volume across nations for the 2,444 publications relevant to PFPS that were located. The number of PFPS research papers, in general, maintained a trend of steady increase from 2000 to 2023, with explosive growth in 2018, witnessing a 40% increase in the number of publications compared to the preceding year, reaching its zenith at 195, and since then the annual number of publications has been maintained at around 165. Overall, the USA commands the highest proportion of both annual and cumulative paper counts, followed by developed countries such as Australia and the United Kingdom. Using a polynomial fit analysis, we found significant correlations between the number of publications and the year of publication (coefficients of determination (R2) for total papers, articles, reviews, and randomized controlled trials were, respectively, 0.8696, 0.9094, 0.9239, and 0.8605). As shown in Figure 2B, the polynomial fit analysis predicts that there will be around 209 publications published in 2025, including 169 original papers, 40 reviews, and 23 RCTs. Generally, there is more research being done at a deeper level as a result of the rise in the fields of sports medicine, orthopedics, and rehabilitation medicine. Yet, it is evident that, despite the annual growth in the number of publications, there is still a scarcity of high-quality RCTs.

### 3.2 Cooperation networks across nations or regions

**Figure 3:**
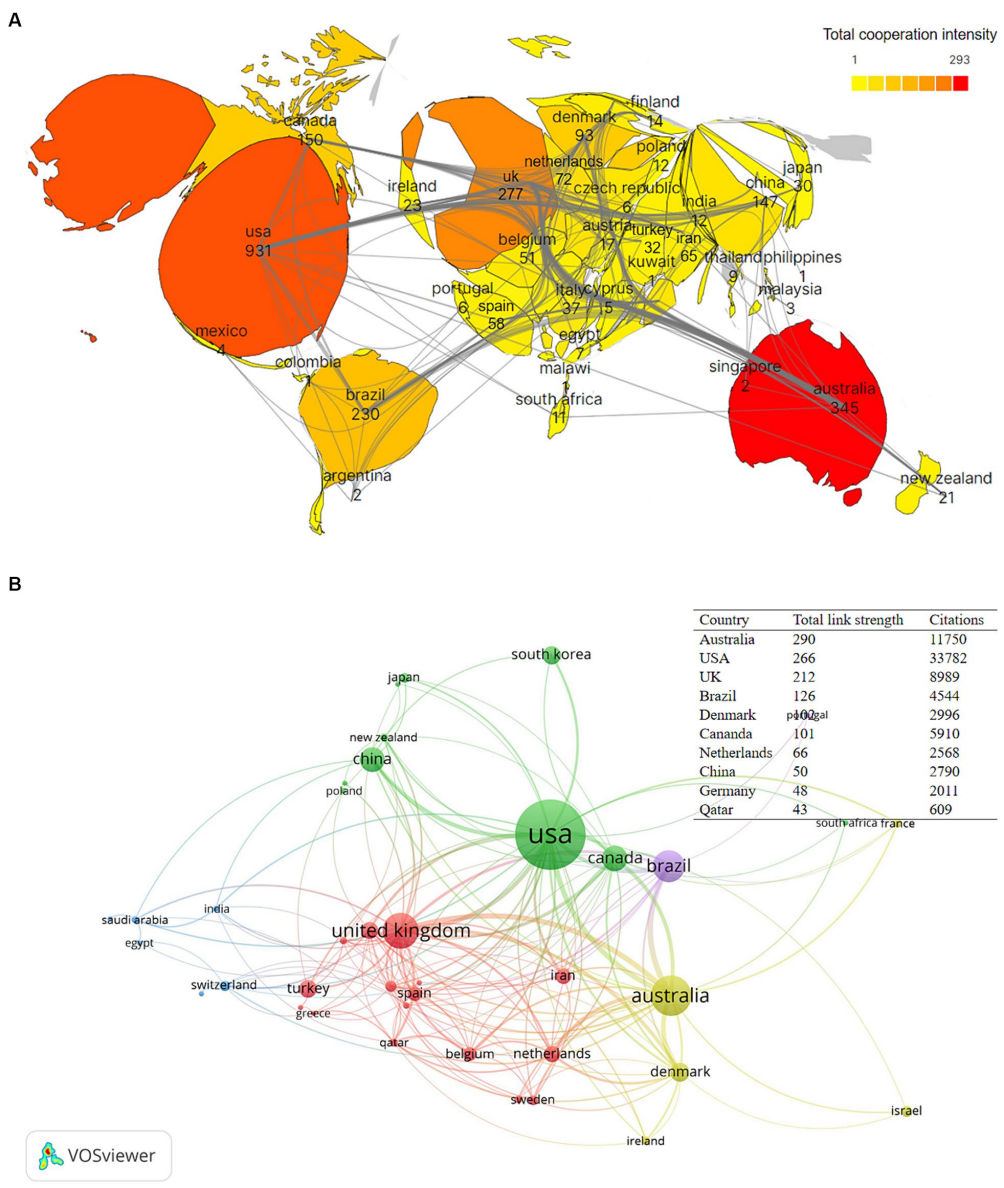
**(A)** Filled chart for the bibliometric analysis of cooperation between countries in the PFPS research field. A larger percentage of country area represents more publications, and approximately darker colors represent stronger collaborative relationships. **(B)** Map of intensity of collaboration by country/region in the PFPS research field. Networks of national/regional cooperation in this field. Circles represent countries and the size of the circle indicates the number of publications. Different colors represent different clusters and connecting lines represent international collaboration between countries. The thickness of the connecting lines indicates the strength of the collaboration, https://tinyurl.com/yqkcy9h6.

The visualization map encompasses a total of 67 countries or regions, with 14 of them having published over 50 articles. Table 1 displays information on the top 10 countries based on the number of publications. Notably, 70% of these top 10 countries are classified as developed nations. Therefore, a substantial disparity persists between advanced and developing countries in this particular research domain. The country with the highest number of publications and the most prominent position in this subject area is the USA (931 articles), which indicates that the USA has the greatest influence in this field. Figure 3A illustrates the international collaboration between the 40 countries/regions that have collaborated on at least five publications. The darker color in the filled Figure 3B represents stronger cooperation with other countries. Therefore, the figure shows that there are good research collaborations between the USA, Australia, and the UK and that North America’s collaborations with Australia are significantly stronger than those with Europe and Asia.

**Table 1:** Top 10 high impact countries and institutions for PFPS research.

Country	Number of articles issued	Institution	Number of articles issued
USA	931	La Trobe Univ	139
Australia	345	Univ Melbourne	106
England	260	Univ Queensland	101
Brazil	230	Univ Wisconsin	96
Canada	150	Aalborg Univ	81
China	99	Univ São Paulo	78
Denmark	93	Univ Calgary	72
South Korea	86	Univ So Calif	64
Turkey	79	Univ Kentucky	60
Germany	73	Univ Fed Sao Carlos	55

### 3.3 Networks of collaboration and research institutes

**Figure 4:**
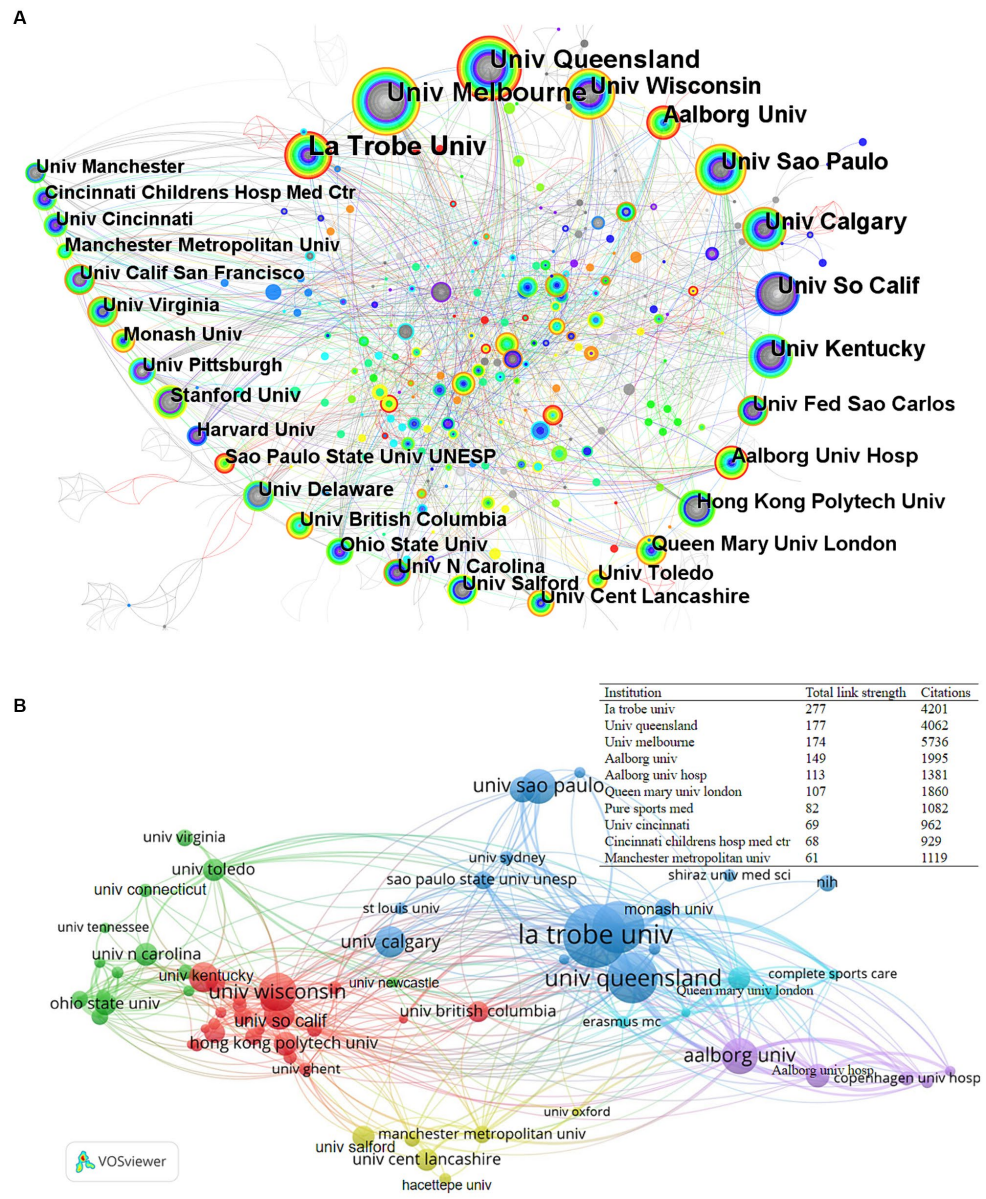
**(A)** Co-occurrence network diagram of institutions for PFPS. Circles in the plot indicate the number of articles, the larger the circle indicates more articles issued by the institution, the thickness of the purple outer circle represents the degree of centrality of the institution, and the connecting line represents the existence of cooperation or co-occurrence relationship. **(B)** Map of the intensity of collaboration among institutions in the PFPS research field. Networks of national/regional cooperation in this field. Circles represent countries and the size of the circle indicates the number of publications. Different colors represent different clusters, while the lines connecting the projects represent international collaboration between countries. The thickness of the connecting lines indicates the strength of the collaboration, https://tinyurl.com/yrsaswak.

A total of 818 institutions published PFPS-related studies, among which 50 institutions had ≥20 articles. The data regarding the top 10 institutions, ranked by the number of published articles, can be found in Table 1 and Figure 4A. The top 10 organizations, which recorded the highest number of published articles, collectively contributed to 34.86% of the overall total of published articles. Figure 4B illustrates the international collaborations between the 70 institutions that have collaborated on at least 12 publications. Three institutions, Ia trobe univ., Univ queensland, and Univ melbourne, have established better research collaboration with some of the other institutions.

### 3.4 Networks of collaborators and high impact authors

**Figure 5:**
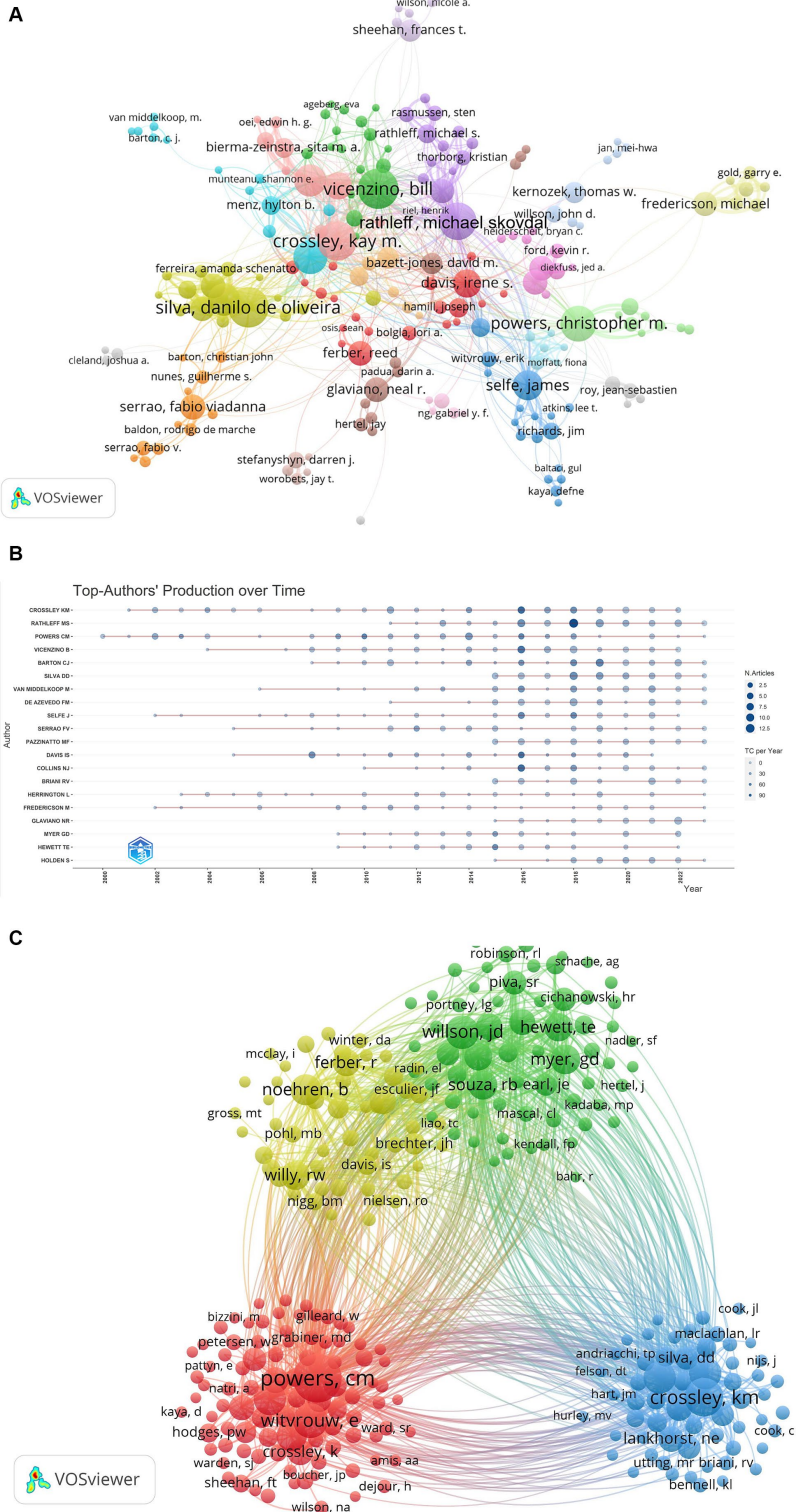
**(A)** Author co-occurrence network map of PFPS. Circles in the plot indicate the number of posts, larger circles indicate more posts by the author, and connecting lines indicate the existence of collaborative or co-occurring relationships, https://tinyurl.com/ypmprb6d. **(B)** Highly prolific authors in PFPS over time. The top 20 prolific researchers in the field and their publications. The larger the node, the more publications. The darker the color, the more citations. The color represents the number of publications and the color represents the number of citations per year. **(C)** Cluster visualization of author co-citation analysis generated based on VOSviewer software. Each node represents an author, and the size of each circle is determined by the author’s co-citation, https://tinyurl.com/ylbl2h5k.

The visualization map incorporated a total of 1,247 authors, with 118 authors having 20 or more publications. Table 2 presents details about the top 10 scholars based on both the number of publications and the number of citations. This information is complemented by Figures 5A,B, there is a certain degree of collaboration among the author teams with each other, which is more obvious among the high-producing authors. The three most prominent researchers in the field of PFPS, i.e., Rathleff MS, Barton CJ, and Crossley KM, with 65, 65, and 63 publications, respectively, are very active and influential authors. Utilizing VOSviewer for author co-citation analysis, the top three authors in terms of co-citation frequency for PFPS research are Powers CM (1,556 times), Crossley KM (888 times), and Barton CJ (609 times), and Figure 5C shows the network of co-cited author relationships, which includes 271 authors with a citation frequency of 50 or above.

**Table 2:** Top 10 high-impact authors of PFPS research.

Author	Number of articles issued (articles)	Country	Author	Frequency of citations	H-index
Rathleff MS	65	Denmark	Powers CM	680	25
Barton CJ	65	Australia	Crossley KM	508	25
Crossley KM	63	Australia	Willson JD	403	13
Vicenzino BT	56	Australia	Witvrouw E	376	12
de Oliveira Silva D	47	Brazil	Souza RB	334	10
Powers CM	42	USA	Barton CJ	320	25
van Middelkoop M	40	Netherlands	Noehren B	313	15
de Azevedo FM	35	Brazil	Taunton JE	304	2
Serrao FV	34	Brazil	Bolgla LA	280	9
Pazzinatto MF	32	Brazil	Boling MC	276	6

### 3.5 Relationships between citations and high impact journals

**Figure 6:**
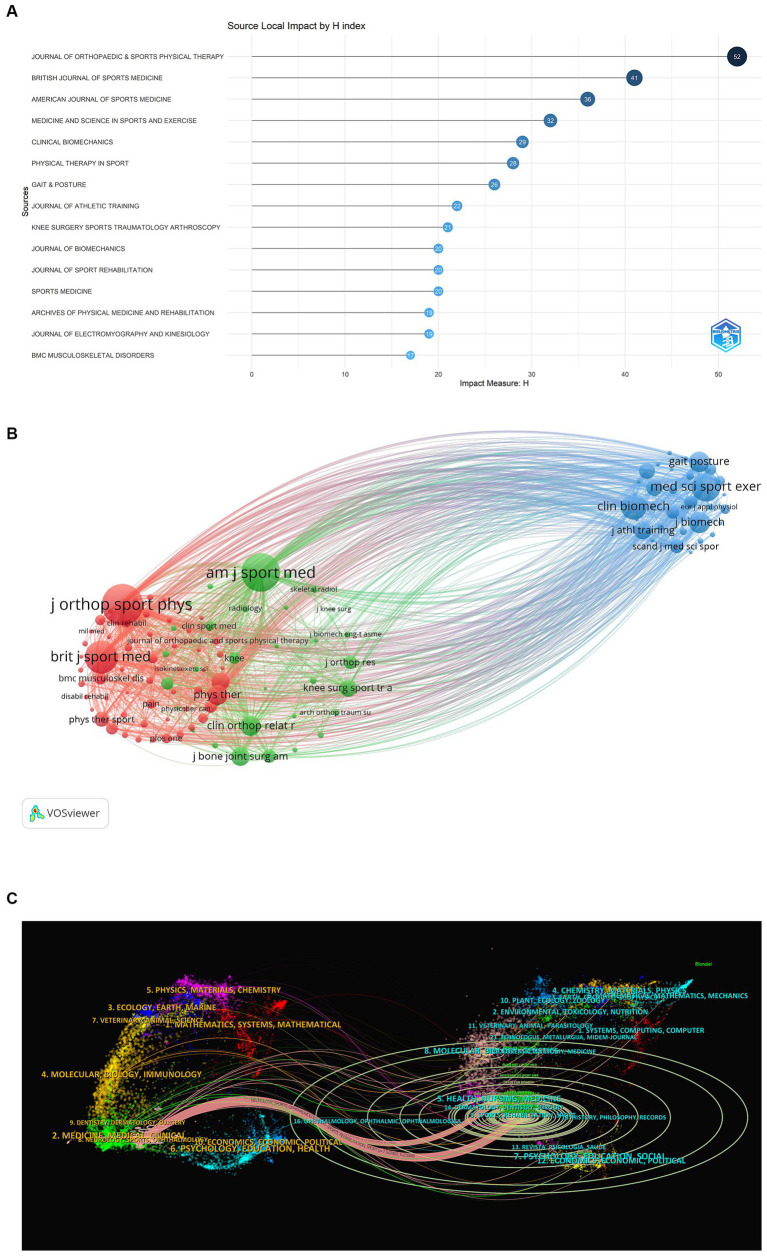
**(A)** H-index of high-impact journals for PFPS research, 2000–2023. **(B)** Cluster visualization of journal co-citation analysis generated based on VOSviewer software. Each node represents a journal and the size of each circle is determined by the co-citation of the journal, https://tinyurl.com/ymneahml. **(C)** Double graph overlay of citing and cited journals in the PFPS research fields. Citing journals on the left, cited journals on the right, the connecting line represents the citation status.

The 2,444 documents retrieved were from 322 journals, and the top three journals in terms of number of publications were JOURNAL OF ORTHOPAEDIC AND SPORTS PHYSICAL THERAPY (138 articles, total citations 4,769, average citations 34.56), PHYSICAL THERAPY IN SPORT (112 articles, total citations 782, average citations 6.98), and GAIT AND POSTURE (102 articles, total citations 923, average citations 9.05). 782 total citations, 6.98 average citations and GAIT AND POSTURE (102, 923 total citations, 9.05 average citations). The high H-index journals in this field of research, with the top three H-indexes are JOURNAL OF ORTHOPAEDIC AND SPORTS PHYSICAL THERAPY (20), BRITISH JOURNAL OF SPORTS MEDICINE (21), AMERICAN JOURNAL OF SPORTS MEDICINE (22); based on the journal co-citation analysis based on VOSviewer, the top three co-cited journals for the PFPS study were JOURNAL OF ORTHOPAEDIC AND SPORTS PHYSICAL THERAPY (7,746 times), AMERICAN JOURNAL OF SPORTS MEDICINE (6,882 times), and BRITISH JOURNAL OF SPORTS MEDICINE (5,130 times), see Figure 6A and Table 3. Figure 6B shows the network of co-cited journal relationships, which includes 123 journals with a citation frequency of 100 or more. Presently, journals publishing articles in this research area exhibit a high impact. The colored paths in the biplot overlay between clusters of journals indicate citation relationships, revealing the trajectories of citations and the flow of knowledge between the citing and cited journals (23). The colored paths indicate that studies published in NEUROLOGY, SPORTS, OPHTHALMOLOGY journals typically cite studies published in SPORTS, REHABILITATION, SPORT and HEALTH, NURSING, MEDICINE. Additional details about the representative citing and cited journals in each cluster can be explored in Figure 6C. For example, the most representative journals in the sports/rehabilitation/exercise cluster are Journal of Orthopaedic and Sports Physical Therapy, American Journal of Sports Medicine, British Journal of Sports Medicine, Medicine and Science in Sports and Exercise, and Clinical Biomechanics.

**Table 3:** Current status of high volume journals for PFPS research.

Journal name	Total literature	Total number of applications	Average number of citations	IF (2023)	JCR (2023)	H-index
JOURNAL OF ORTHOPAEDIC AND SPORTS PHYSICAL THERAPY	138	4,769	34.56	6.1	Q1	52
PHYSICAL THERAPY IN SPORT	112	782	6.98	2.4	Q2	28
GAIT AND POSTURE	102	923	9.05	2.4	Q2	26
JOURNAL OF SPORT REHABILITATION	86	491	5.71	1.7	Q3	20
BRITISH JOURNAL OF SPORTS MEDICINE	74	3,077	41.58	18.4	Q1	41
CLINICAL BIOMECHANICS	74	1,470	19.86	1.8	Q3	29
AMERICAN JOURNAL OF SPORTS MEDICINE	70	2,121	30.30	4.8	Q1	36
JOURNAL OF ATHLETIC TRAINING	65	689	10.60	3.3	Q2	22
KNEE	63	252	4.00	1.9	Q3	14
BMC MUSCULOSKELETAL DISORDERS	54	405	7.50	2.3	Q2	17

## 4 Keyword visualization analysis

### 4.1 Keyword co-occurrence clustering analysis of research hotspots

**Figure 7:**
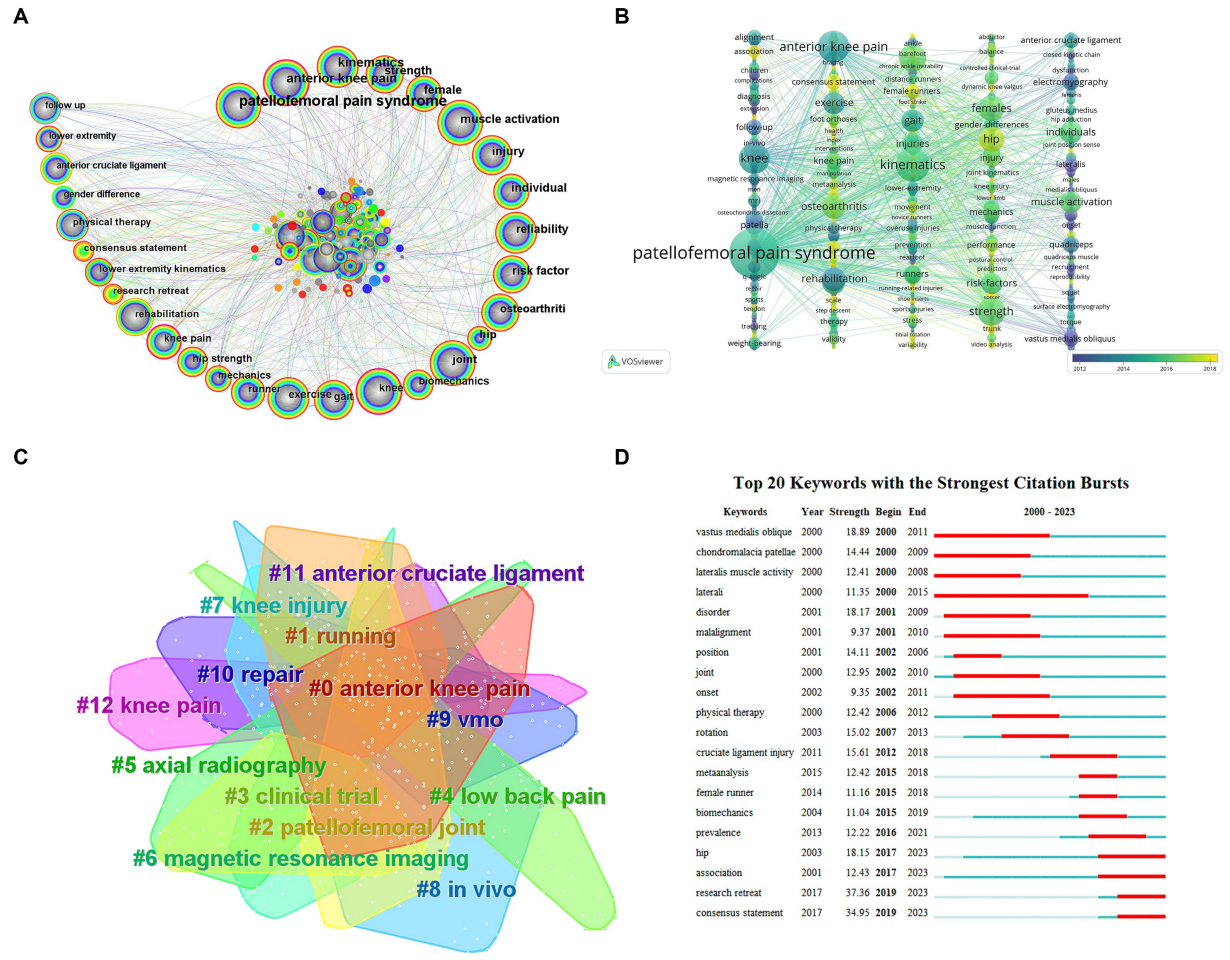
**(A)** Keyword co-occurrence graph of PFPS. The circles in the figure represent keywords, the larger the circle the higher the frequency of the keyword. Darker to lighter colors represent the years from far to near, the connecting line represents the connection between keywords, and the thickness of the purple outer circle represents the centrality of the keywords. **(B)** VOSviewer-based time view of PFPS keywords. **(C)** Keyword co-occurrence clustering map for PFPS. Dark to light colors represent years from far to near, and connecting lines represent links between keywords. **(D)** Keyword emergence graph of PFPS. “▃” in the graph is the year in which the emergent word appeared and persisted, in chronological order from top to bottom.

When summarizing research hotspots and investigating research trends, keyword analysis is pivotal (24). High co-occurrence keywords are shown in Figures 7A,B, as well as Table 4, which has 570 nodes overall and 3,039 connections. High centrality and frequency keywords highlight the most active areas of research during that time. The clustering map of keyword co-occurrences in this domain is shown in Figure 7C. A total of 14 clusters were formed through the conventional LLR approach. Moreover, the term clustering analysis indicated that as the aggregation level increased, the homogeneity among studies also increased (25). The biggest cluster was designated as #0, and so on, with the cluster size and cluster number being negatively related. As can be seen from the figure the clusters are all closely interlocked and linked to each other. Keyword co-occurrence and cluster analysis yielded that anterior knee pain, lower extremity kinematics and biomechanics, hip-knee and core strength, females (runners), muscle activation (vastus medialis oblique), risk factors, and imaging are current research hotspots in this area.

**Table 4:** PFPS high frequency keywords and centrality TOP10.

Keywords	Frequency	Keywords	Centrality
Patellofemoral pain syndrome	1,528	Knee	0.16
Anterior knee pain	546	Anterior knee pain	0.12
Kinematics	477	Patellofemoral joint	0.12
Strength	386	Knee pain	0.1
Female	317	Follow up	0.1
Muscle activation	301	Anterior cruciate ligament	0.09
Injury	298	Strength	0.08
Individual	253	Electromyographic activity	0.08
Reliability	250	Motion	0.08
Risk factor	245	Female	0.07

### 4.2 Study of modern research trends via emergent word emergence

The 20 most common terms are those that have been filtered from the WOS database and indicate keywords that have been regularly referenced throughout time, displaying trends and hotspots. The most frequent word was “research retreat” (37.36), followed by “consensus statement” (34.95), and in third place was “vastus medialis oblique” (18.89). The terms “consensus statement” (2019–2023), “research retreat” (2019–2023), “association” (2017–2023), and “hip” (2017–2023) have experienced more frequent mutations throughout the previous 5 years and have continued to do so, Figure 7D. We may gain a general understanding of the research hotspots and anticipated developments in this sector by combining the temporal dynamics of keywords and Burst analysis. The emergent words consensus statement, research retreat, association and hip will remain hot and become research trends.

## 5 Visual analysis of key documents

### 5.1 Key literature analysis of research hotspots

**Figure 8:**
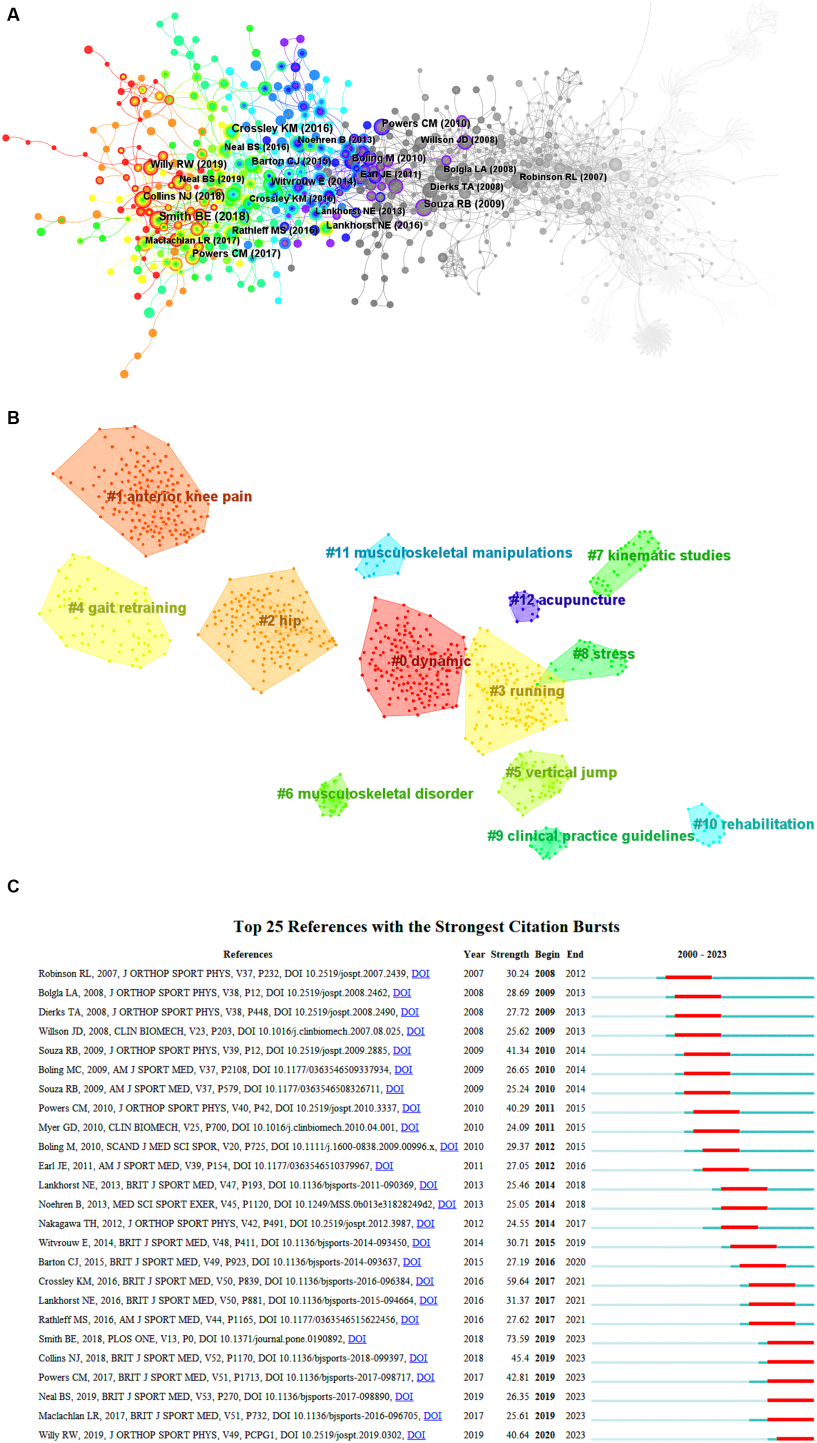
**(A)** Network diagram of co-cited literature in PFPS. Circles in the figure represent co-citations (showing the first author’s name), with larger circles representing more citations. The connecting line represents the co-citation situation among the literature, and the thickness of the purple outer circle represents the centrality of the literature. **(B)** Co-occurrence clustering plot of PFPS key literature. Light to dark colors represent years from far to near, and connecting lines represent links between keywords; this figure is arranged chronologically from light to dark (left to right) cluster colors. **(C)** Key literature emergence graph of PFPS. “▃” in the figure is the year in which the emergent citation appeared, and “▃” in the figure is the node where the citation of the emergent citation suddenly rises, in chronological order from top to bottom.

This region yielded 2,444 papers in total, with a total citation frequency of 9,307. The top 10 highly cited publications are detailed in Figure 8A and Tables 5, 6. The literature that provides a significant theory or novel idea on the subject is typically referred to as the key node paper. It is also the paper that is most likely to lead to the creation of new research hotspots. Through the analysis of co-citations involving main nodes and highly read publications, the research centers and evolutionary trajectories of the PFPS field can be categorized into three main groups: clinical studies, consensus statements or clinical guidelines, and meta-analyses or general reviews. The 1st, 2nd, 3rd, 7th, 8th, and 10th items in centrality, and the 2nd, 3rd, 7th, 8th, and 9th items in citation frequency are clinical studies, the 9th item in centrality, and the 1st, 5th, and 6th items in citation frequency are consensus statements or clinical guidelines, the 4th and 6th items in centrality and the 4th item in citation frequency are meta-analyses, and the 5th item in centrality and the 10th item in citation frequency are general review categories. The clinical studies widely cited literature involved intensive interventional therapy for proximal hip joint (center degree 1st, 2nd, 3rd, 4th, 5th, 6th, and cited frequency 3rd, 8th studies), biomechanical perspective studies (center degree 8th, 10th, and cited frequency 2nd studies), consensus statements or guidelines (center degree 9th and cited frequency 1st, 5th, 6th studies), epidemiological survey studies (cited frequency 4th, 7th studies), prognostic studies (centrality 7th and cited frequency 9th studies), and injury factor studies (cited frequency 10th studies).

**Table 5:** Top 10 PFPS co-citation centrality rankings.

Author	Title	Centrality	Year	Journal
Khayambashi K (26)	The effects of isolated hip abductor and external rotator muscle strengthening on pain, health status, and hip strength in females with patellofemoral pain: a randomized controlled trial	0.2	2012	J Orthop Sports Phys Ther
Fukuda TY (27)	Short-term effects of hip abductors and lateral rotators strengthening in females with patellofemoral pain syndrome: a randomized controlled clinical trial	0.18	2010	J Orthop Sports Phys Ther
Khayambashi K (28)	Posterolateral hip muscle strengthening vs. quadriceps strengthening for patellofemoral pain: a comparative control trial	0.18	2014	Arch Phys Med Rehabil
Nascimento LR (29)	Hip and knee strengthening is more effective than knee strengthening alone for reducing pain and improving activity in individuals with patellofemoral pain: a systematic review with meta-analysis	0.16	2018	J Orthop Sports Phys Ther
Heiderscheit BC (30)	Lower extremity injuries: is it just about hip strength?	0.15	2010	J Orthop Sports Phys Ther
Lack S (31)	Proximal muscle rehabilitation is effective for patellofemoral pain: a systematic review with meta-analysis	0.13	2015	Br J Sports Med
Lankhorst NE (32)	Factors that predict a poor outcome 5–8 years after the diagnosis of patellofemoral pain: a multicentre observational analysis	0.12	2016	Br J Sports Med
Willson JD (33)	Lower extremity mechanics of females with and without patellofemoral pain across activities with progressively greater task demands	0.12	2008	Clin Biomech
Crossley KM (34)	2016 Patellofemoral pain consensus statement from the 4th International Patellofemoral Pain Research Retreat, Manchester. Part 2: recommended physical interventions (exercise, taping, bracing, foot orthoses, and combined interventions)	0.11	2016	Br J Sports Med
Salsich GB (35)	Do females with patellofemoral pain have abnormal hip and knee kinematics during gait?	0.1	2010	Physiother Theory Pract

**Table 6:** Top 10 ranking of co-citation frequency of PFPS.

Author	Title	Cited frequency	Year	Journal
Crossley KM (1)	2016 Patellofemoral pain consensus statement from the 4th International Patellofemoral Pain Research Retreat, Manchester. Part 1: Terminology, definitions, clinical examination, natural history, patellofemoral osteoarthritis, and patient-reported outcome measures	145	2016	Br J Sports Med
Powers CM (36)	The influence of abnormal hip mechanics on knee injury: a biomechanical perspective	96	2010	J Orthop Sports Phys Ther
Souza RB (37)	Differences in hip kinematics, muscle strength, and muscle activation between subjects with and without patellofemoral pain	88	2009	J Orthop Sports Phys Ther
Smith BE (7)	Incidence and prevalence of patellofemoral pain: a systematic review and meta-analysis	82	2018	PLoS One
Witvrouw E (38)	Patellofemoral pain: consensus statement from the 3rd International Patellofemoral Pain Research Retreat held in Vancouver	77	2014	Br J Sports Med
Barton CJ (24)	The “Best Practice Guide to Conservative Management of Patellofemoral Pain”: incorporating level 1 evidence with expert clinical reasoning	65	2015	Br J Sports Med
Boling M (4)	Gender differences in the incidence and prevalence of patellofemoral pain syndrome	65	2010	Scand J Med Sci Sports
Earl JE (25)	A proximal strengthening program improves pain, function, and biomechanics in women with patellofemoral pain syndrome	63	2011	Am J Sports Med
Lankhorst NE (32)	Factors that predict a poor outcome 5–8 years after the diagnosis of patellofemoral pain: a multicentre observational analysis	63	2016	Br J Sports Med
Lankhorst NE (22)	Factors associated with patellofemoral pain syndrome: a systematic review	62	2013	Br J Sports Med

### 5.2 Co-occurrence clustering of important literature is used to analyze research hotspots

Cluster analysis can pinpoint subfields as significant research hotspots by examining co-cited literature (39). The average cluster profile value (Silhouette: S) is 0.8459, surpassing 0.5, suggesting a robust cluster. Additionally, the clustering module value (Modularity, Q) is 0.9308, exceeding 0.5, indicating a noteworthy cluster structure (40). A total of 13 clusters were created using the traditional LLR algorithm, and a high degree of aggregation was found in the key literature when the clusters were analyzed. The literature clustering unveiled the more prominent sub-topic directions of research in the field, and there was an inverse relationship between cluster size and cluster number, with #0 being the largest cluster and so forth, Figure 8B. We can conclude that the top research themes in this field are dynamics, anterior knee pain, hip, running, gait retraining, musculoskeletal disorders, kinesiology research, stress, clinical practice guidelines, rehabilitation, musculoskeletal manipulation and acupuncture. From the clustering emergence time, the future research trend themes in this field are running, gait retraining, hip, kinesiology research and rehabilitation.

### 5.3 Research frontier trends based on the appearance of important publications

Of the 25 citations with the highest emergent intensity screened against the WOS database, the highest emergent intensity was located in Smith et al. (7) in 2018, which found a high incidence and prevalence of PFPS (73.59), followed by Crossley et al. (1) in 2016 for his fourth consensus statement on patellofemoral pain, which focused on terminology, definitions, clinical examination, natural history, patellofemoral pain, and patient-reported outcomes (59.64); in third place was Collins et al. (41) published in 2018 the recommendations of the 5th International Patellofemoral Pain Research Refresher, a consensus statement on exercise therapies and physical interventions (orthoses, tapes, and manipulative therapy) for the treatment of patellofemoral pain (45.40), Figure 8C. Based on the literature emergence it is possible to divide the hot spots of research in this field into four parts in chronological order, with the first part focusing on hip and knee muscle strength in female patients with PFPS (33, 42) and studies analyzing the kinematic characteristics of the lower limbs during different activity tasks (running, stairs; 2007–2011) (21, 37); the second part focuses on the investigation of gender variations in PFPS incidence and prevalence (4, 43) and elements that increase the risk of PFPS development (2012–2015) (22, 44); the third part focuses on consensus statements or clinical guideline studies of PFPS (24, 38) and studies on factors affecting poor prognosis of PFPS (2016–2018) (32, 45); the fourth part focuses on studies in which exercise interventions with proximal strengthening are effective in improving PFPS (46), network meta-analysis (7), research refresher or clinical practice guidelines (41, 47, 48) and psychological characteristics of adolescent patients (2019–present) (49). The emergent intensity of some of the emergent citations has persisted to date, additionally, based on reviewing highly emergent citations, it is foreseeable that upcoming research trends may be dominated by studies on risk factors for poor prognosis in PFPS, studies on exercise interventions targeting external rotation of the hip to improve PFPS, network meta-analyses, clinical practice guidelines, and research fellowships in this field, and studies related to psychological characteristics of adolescent patients.

### 5.4 Using thematic maps to analyze research hotspots and cutting-edge trends

**Figure 9:**
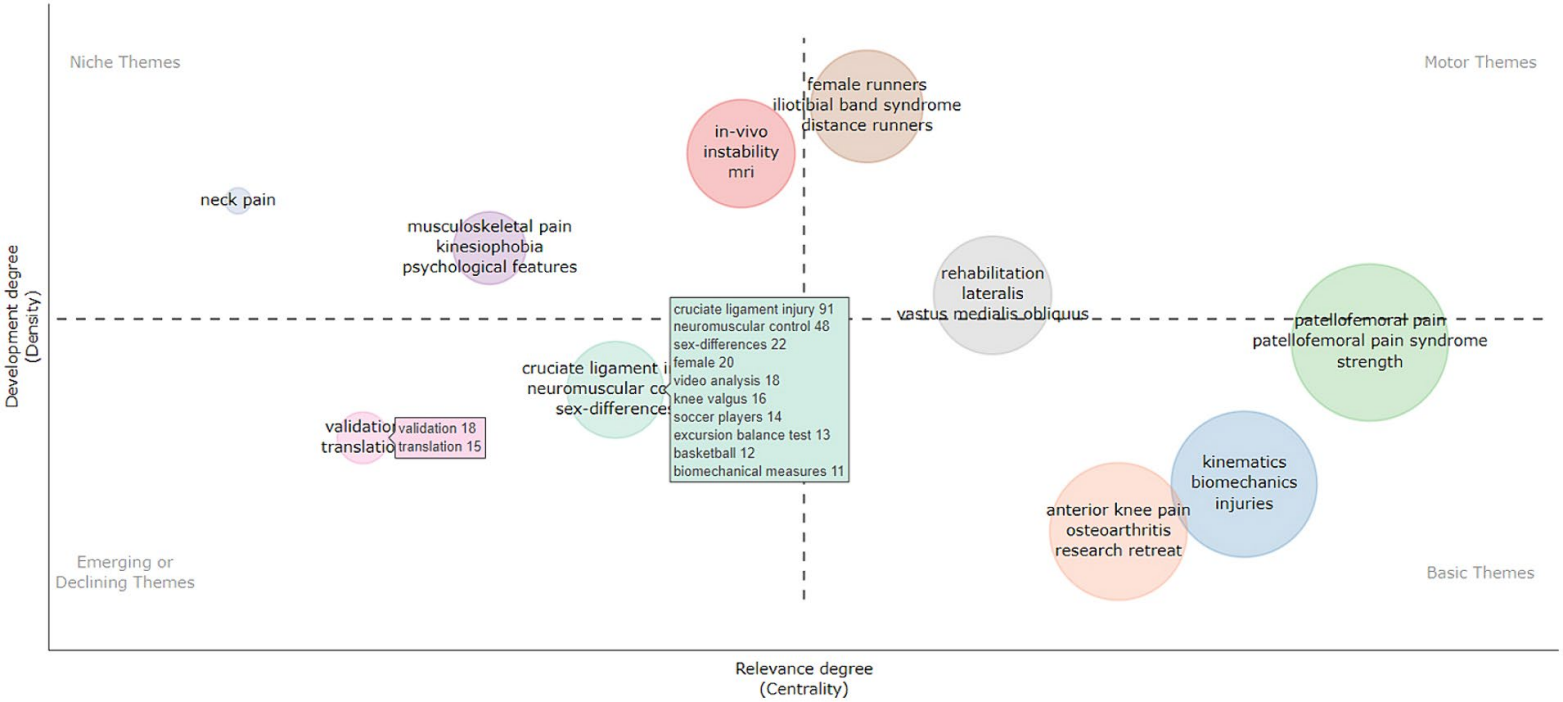
Thematic maps of PFPS.

The R-Bibliometrix software was used to construct the theme maps, which are presented as a two-dimensional matrix. Figure 9 illustrates the two dimensions of matrix centrality on the x-axis and density on the y-axis. It can be inferred from the quadrant where the important bubbles are situated that female runners, iliotibial tract syndrome, and lateral and medial femoral muscles are the hotspots of research in this area, while cruciate ligament injury, neuromuscular control, gender differences, validation, and transformation themes might advance or end in the future, while in kinesiology, biomechanics, osteoarthritis, and research refresher theme directions need more in-depth research in the future.

## 6 Discussion

Over the past 23 years, PFPS research has received a great deal of attention. This phenomenon may be related to the growing number of sports enthusiasts and their increasing economic burden, as well as the popularization of the concept of “exercise as medicine.” In this study, we conducted a comprehensive bibliometric analysis of PFPS research from 2000 to 2023. In the last 23 years, a total of 2,444 papers with 9,307 citations were published worldwide, maintaining an overall trend of gradual increase. These results indicate the continued attention and interest of researchers in this field.

A total of 64 countries or regions are involved in research in this field, with the average year of publication of academic publications from the USA, Australia, the United Kingdom, and Brazil being relatively early. They have made outstanding contributions in the early stage of research in this field, laying a solid knowledge base for subsequent research. Of the top 10 countries in terms of annual publications, the USA had more annual publications than all other countries combined until 2008. Since then, the USA share of annual publications has gradually declined. The USA has made outstanding contributions in this field, with the highest number of articles and citation frequency, indicating a high level of research, widely recognized and cited academic results by many scholars, and high academic influence. The most frequently cited study, by reviewing biomechanical and clinical studies in this field, found that impaired muscle control of the hip, pelvis, and trunk affects the kinematics and dynamics of multiple planes of the tibiofemoral and patellofemoral joints. It was shown that impaired hip motion is the cause of injuries such as patellofemoral joint pain, ACL tears, and iliotibial bundle syndrome (36). Australia, although only ranked second in terms of publication output, had the highest total link strength, indicating extensive collaborations with other countries/regions, in particular good research collaborations between the USA, UK, Brazil, and Denmark, with North America’s partnership with Europe being significantly stronger than that with Asia and South America. It is also worth noting that 60% of the top 10 countries/regions with the highest number of publications are located in North America and Europe, 20% are located in East Asia, and the remaining 20% are located in South America and Australia, respectively, suggesting that they are the main strongholds for this research area.

La Trobe Univ Australia has become the leading institution in the field with the largest number of publications. These publications have made a significant contribution to early research in the field and on average were published relatively early. It also has the highest total link strength, which indicates a high level of collaboration with other institutions, particularly with Univ Melbourne, Univ Queensland, Aalborg Univ, Univ São Paulo, and Queen Mary Univ London. One of these highly cited papers was an evidence-based framework consensus proposing a pathomechanical model of patellofemoral pain, which placed known correlates in the context of a pathomechanical model of patellofemoral pain and proposed that patellofemoral pain is associated with abnormal loading of the patellofemoral joint (elevated joint stress) (47). The University of Melbourne, Australia, not only ranked high in terms of publication output but also had the highest number of citations, indicating a high level of research, academic results widely recognized and cited by many scholars, and high academic impact. One of the highly cited articles is the study of outcome indicators for the treatment of PFPS [Three 10-cm visual analog scales (VASs) for usual pain (VAS-U), worst pain (VAS-W), and pain on 6 aggravating activities (VAS -activity); the Functional Index Questionnaire (FIQ); the Anterior Knee Pain Scale (AKPS)] for retest reliability, validity, and responsiveness, and the study determined that the AKPS and VAS are reliable, valid, and responsive indicators of outcome (50). The top 10 organizations in terms of the number of publications accounted for 34.86% of the total number of publications. This implies that they have achieved considerable academic success. However, the collaboration among these institutions is not significant compared to the collaboration among countries. Collaboration helps researchers in the field to exchange ideas and resources, which is essential for further research development.

Among the top 10 authors in terms of the number of publications, authors from developed countries and developing countries accounted for 60% and 40%, respectively, with Rathleff MS from Aalborg Univ, Denmark, and Barton CJ from La Trobe Univ, Australia, being the most prolific authors in the field together, which indicates that they have significant academic influence and important contributions in the field. The research team represented by Rathleff MS and others focused on the presence of localized, distal nociceptive hypersensitivity in adolescent females with PFPS (51) and impaired conditioned pain regulation (20) Consensus statements on exercise therapy and physical interventions for the treatment of PFPS (34, 41). The research team represented by Barton CJ focuses on foot posture, kinematics, and biomechanical characterization during gait in patients with PFPS (52, 53), as well as clinical efficacy studies focusing on different exercise intervention modalities with a focus on activation of the gluteal muscles (54, 55). Powers CM is not only the sixth author in terms of number of publications, but also the first author in terms of citation frequency and H-index, indicating that the research has a high academic impact and the academic results are widely recognized and cited by many scholars. The most frequently cited study was from a biomechanical perspective, confirming through a retrospective study that proximal lower extremity strengthening measures can be beneficial to patients with various knee disorders, and categorizing proximal strengthening training into pelvic and trunk stability and dynamic hip control, and that females are more susceptible to the effects of neighboring joints than males (36). Notably, Brazilian scholars accounted for 40% of the top 10 publications, with de Oliveira Silva D from Univ São Paulo, Cuba, being the most representative, interacting closely with Pazzinatto MF and de Azevedo FM, and focusing on the effects of altered lower limb biomechanics on pain and function in patients with PFPS, such as during stair climbing Decreased knee flexion (56) or vertical ground reaction forces (57) Altered effects on PFPS, Q-angle kinematic and static measurements (58) or foot exostosis kinematic measurements (59) reliability studies for the diagnosis of patellofemoral pain. There is also a focus on novel ways to provide educational and exercise intervention treatments for patients with PFPS (60, 61).

The top 10 journals in terms of number of publications accounted for 34.29% of the total publications, and only these two journals among the top 10 journals in terms of number of publications had IF > 5 points. These two journals accounted for 25.30% of the total number of publications in the top 10 journals. Therefore, it is challenging to publish in high-impact factor journals. Journal of Orthopaedic and Sports Physical Therapy is the number one journal in the field in terms of number of publications, total citations, and H-index. A high-profile paper found that female PFPS patients had increased hip internal rotation during functional tasks (running, drop jump, step-down) accompanied by decreased hip muscle strength and increased gluteus maximus EMG signal intensity. Indicating that patients attempt to stabilize the hip joint by activating the gluteus maximus muscle, highlighting the importance of hip stability in PFPS (37). The British Journal of Sports Medicine is the Q1 journal with the highest IF and the top-ranked journal in terms of average number of citations. Its most cited paper was the publication of the fourth consensus statement on PFPS, where the first part of the main common statement clarified the terminology, definitions, clinical examination, natural history, patellofemoral pain, and patient-reported outcome measures in the field (1). The second part, recommending physical interventions, advocated six treatment recommendations, namely exercise (especially combined hip and knee strengthening), taping, bracing, foot orthotics, and combined interventions (34). Journal of Orthopaedic and Sports Physical Therapy is the Q1 journal with the highest H-index and the British Journal of Sports Medicine is the highly cited Q1 journal with the highest IF. This indicates that these two journals are highly recognized and authoritative and their published articles are of great academic reference value. When publishing research in this field, you can prioritize submitting to these highly productive journals, and when searching for relevant literature, you can prefer the proceedings of these highly cited journals.

Highly cited literature and high centrality literature typically reflect high-quality research and scholarly impact, while also providing insights into the major research foci in a given field (62). Therefore, analyzing the highly cited and highly centered literature can provide a preliminary understanding of research trends and directions in the field. Current research suggests that weak hip muscles lead to increased femoral adduction and medial rotation during dynamic weight-bearing activities, which increases lateral patellofemoral joint compressive stresses and leads to patellofemoral plane overload, which in the long term will result in patellofemoral joint pain (63). High-impact scholars, led by Khayambashi et al. (26), have found that 8 weeks of isolated hip abductor-external rotator strengthening training was effective in improving pain, health status, and hip strength in female patients. Fukuda et al. (27) found that a 4-week short-term hip and knee joint strengthening program was more effective in reducing pain when walking downstairs in sedentary female patients. Similar studies such as Lack (31) found that selecting an open-chain exercise approach to strengthen the proximal limb was highly significant in improving pain function in patients. Souza et al. (37) found an increase in hip internal rotation in female patients during functional tasks accompanied by a decrease in hip muscle strength and an increase in EMG signal intensity in the gluteus maximus by collecting surface EMG signals. This suggests that there is increased activation of the gluteus maximus in an attempt to recruit weak muscles to stabilize the hip joint. Earl et al. (25) found that strengthening of the core muscles was equally effective in improving symptoms. Nascimento et al. (29) similarly showed in a Meta-analysis that included 673 patients from 14 studies that combined hip and knee strengthening was more effective than knee strengthening alone in reducing pain and improving activity in patients with patellofemoral pain. Meanwhile, a Meta-analysis by Lack et al. (31) similarly demonstrated that proximal combined quadriceps rehabilitation reduces pain and improves function in the short-term (3 months), medium-term (1 year), and long-term (>1 year), highlighting the long term value of treating PFPS. Heiderscheit et al. (30) concluded that treatment and prevention of PFPS should be multifaceted and that in addition to lumbopelvic and hip stability, other factors such as patellar alignment, quadriceps strength, foot posture, and beliefs about avoiding fear should be taken into account. In terms of the biomechanical characteristics of the lower extremities in patients with PFPS, the etiology and progression of the disease are frequently associated with excessive patellofemoral joint compressive stress. Weakness of the peripatellar muscle groups may adversely affect the mechanics of the tibiofemoral and patellofemoral joints in multiple planes (36). Indeed, the location of patellofemoral joint pathology is most common in the region of the patellofemoral joint surface associated with maximal loading. Therefore, it is particularly important to biomechanically analyze the distribution of stresses on the patellofemoral joint during motion. Scholars, led by Willson et al. (33), have found that patients do exhibit increased external rotation of the knee, decreased internal rotation of the knee, increased hip adduction, and decreased internal rotation of the hip in activities of varying difficulty such as single-leg squatting, running, and repetitive one-legged jumps. Salsich et al. (35) found that female patients had less hip adduction in the early stance phase of brisk walking and more hip adduction in the later stance phase, with more severe patients exhibiting additional features of knee valgus. Powers et al. (36) concluded from a biomechanical point of view in a retrospective study that proximal lower extremity strengthening measures can be beneficial for patients with a variety of knee disorders, and categorized proximal strengthening training into pelvic and trunk stability and dynamic hip control, and that women are more susceptible than men to adjacent joints. Consensus statements and guidelines in the field of PFPS have been actively improved to promote a consensus on systematic specifications for clinicians and researchers to better evaluate and manage patients. Represented by the highest frequency of citations, Crossley et al. (1) published the fourth consensus statement on patellofemoral pain syndrome, in which the first part of the main common statement clarifies the terminology, definitions, clinical examination, natural history, patellofemoral pain, and patient-reported outcomes in the field. The second part, recommending physical interventions, advocated six treatment recommendations, namely exercise (especially in combination with hip and knee exercises), taping, bracing, foot orthotics, and combined interventions (34). Meanwhile, Witvrouw et al. (38) published the third patellofemoral pain syndrome Consensus Statement focusing on the development of consensus in three specific areas, natural history and local (knee region) factors affecting patellofemoral pain, trunk and distal factors affecting patellofemoral pain, and rehabilitative innovations for patellofemoral pain. In addition, Barton et al. (24, 64) focused on best practice guidelines for conservative treatment, which are individualized and tailored multimodal intervention programs that include gluteal and quadriceps strengthening, patellar intramuscular efficacy tapes, and an emphasis on education and activity modification. In terms of the incidence and prevalence of PFPS, Smith et al. (7) found that approximately one in 10 military recruits, one in 14 adolescents are suffering from pain, and one in five of the general population experience patellofemoral joint pain. Meanwhile, Boling et al. (4) found that at the US Naval Academy, the prevalence was 2.23 times higher in women than in men. Lankenberg et al. In terms of treatment prognosis, Lankhorst et al. (32) found that more than 50% of patients developed chronic pain 5–8 years after initial treatment and suggested that longer-lasting pain and worse functional scores portend a poor prognosis. The study was ranked in the top 10 for both high citations and high centrality, highlighting the importance of improving the prognosis of PFPS treatment. In terms of PFPS injury factor studies, Lankhorst et al. (22) conducted a meta-analysis based on 47 publications, which summarized that greater Q angle, femoral groove angle, and patellar tilt angle, less hip abduction strength, lower peak knee extension torque, and less hip external rotation strength were associated with patellofemoral joint pain.

In recent years, the effectiveness of treating PFPS with combined hip and knee interventions has become well-known. There have been several studies mentioning new exercise interventions for the treatment of PFPS, such as blood flow restriction training interventions, gait retraining interventions, and neuromuscular control training interventions. Blood flow restriction (BFR) training involves the use of a proximal limb compression cuff to inflate and pressurize skeletal muscle, causing ischemia and hypoxia by restricting blood flow. Numerous studies have shown that BFR combined with low-intensity resistance training can increase muscle strength and reduce muscle atrophy (65). A meta-analysis that included nine studies reported that BFR had a positive effect on increasing strength, altering muscle size, and improving athletic performance in healthy athletes (66). Constantinou A randomly assigned 60 patients with PFPS to the combination with a hip and knee strengthening training group and a blood flow restriction training strengthening group, the study found that the improvement in knee function was essentially equivalent in both groups at the end of treatment (67). Giles et al. randomized 79 patients with PFPS into a low-load BFR training group and a conventional quadriceps strengthening training group and found a 93% reduction in pain from activities of daily living in the BFR group after 8 weeks of treatment (68). Gait retraining may be a potential treatment for PFPS. In patients with PFPS who lack abnormal physical examination findings, overactivity in dynamic biomechanics or movement errors during activity should be considered. One study performed gait retraining from the back foot to the front foot in patients with PFPS and found that post-intervention and 1-month follow-up showed improvement in knee pain, and biomechanical changes indicated that gait retraining was successful (69). One study reported an increase in gait speed and a decrease in mean vertical load in the gait retraining group compared with the exercise-only and education-only groups, although there were no clinically significant differences in the final results (70). Currently, there is still a lack of clarity regarding the short- and long-term benefits of gait retraining, which may be attributed to the difficulty of implementing the process, suggesting that standardization of the implementation of normative studies would help to determine its effectiveness. Neuromuscular training is a comprehensive treatment program that includes balance training, strength training, and proprioceptive training. Neuromuscular control training for patients with PFPS includes balance and proprioceptive training of the knee, and strength training of the quadriceps and gluteus muscles. Motealleh et al. (71) found that 4 weeks of neuromuscular control training was more effective than conventional physiotherapy exercises in improving pain, balance, and functional performance in women with PFPS. Also, the use of muscle patches (72) and a patellar brace (73, 74) and foot orthoses (75–77), etc. in conjunction with exercise interventions have also been shown to further improve PFPS symptoms. In addition, lubrication of the joint space, reduction of inter-articular friction, inhibition of the inflammatory response, and repair of cartilage damage can also be achieved by injecting drugs into the joint cavity. Intra-articular fluid injection drugs include nonsteroidal anti-inflammatory drugs (NSAIDs) (78), glucocorticoids, sodium hyaluronate (79), platelet-rich plasma (PRP), and others. Notably, in recent years, as platelet-rich plasma continues to be studied in the fields of orthopedics and sports medicine, more and more scholars have begun to explore the role of PRP in the treatment of chronic knee pain. Some studies have concluded that PRP injection for PFPS is superior to other injections in terms of pain reduction and functional improvement in short-term follow-ups (80). Currently, a variety of platelet-rich plasma-loaded sustained-release systems, including injectable hydrogel microspheres (81) injectable hydrogel formulations, etc. (82), and the multi-point injection Peppering technique (83), have been successfully developed to achieve prolonged localized action of PRP. However, there is a lack of research related to the application of PFPS.

Co-occurrence and clustering analysis of keywords and key literature indicated hip-knee joint strength and core strength, lower extremity kinematics, and biomechanics, females (runners), muscle activation, risk factors, gait retraining, clinical practice guidelines, and rehabilitation as the hot keywords for research in this area. The analysis of clustered timeline plots of keywords and key literature indicated exercise therapy, kinesiology and biomechanics, running injuries, gait retraining, imaging, risk factors for poor prognosis, dynamic knee valgus (hip adduction and internal rotation), clinical practice guidelines and research advancement, quality of life, osteoarthritis and network meta-analysis as the keywords of research trends in this field. These research hotspots not only reflect current trends but also provide valuable insights for future research. Specifically, the main research hotspots are the clinical efficacy of combined hip and knee strength strengthening to improve PFPS, lower extremity kinematic and biomechanical characterization, gait retraining, risk factors, clinical practice guidelines, and the application of network meta-analysis. In the future, the clinical efficacy of novel exercise therapies (blood flow limiting training interventions, gait retraining interventions, and neuromuscular control training interventions) to improve PFPS, gait retraining for runners (especially females) to prevent PFPS, and novel PRP formulations for the treatment of PFPS can be further investigated.

## 7 Limitations of the study

This analysis focused exclusively on English literature related to PFPS in the SCIE core dataset of the Web of Science database. As a result, valuable literature from other databases or in other languages may not have been included, with some limitations in literature searching. In the process of generating visualization maps, there is no standardized setting process for time partitioning, screening criteria (threshold), and pruning method for the time being. Different time partitions result in inconsistencies in the number of documents in the partitioned time zones. The screening criteria lead to inconsistency in the number of extracted literature with the highest number of citations in each time partition after segmentation. The pruning method leads to inconsistency in the way the research network is pruned using the path-finding algorithm, so there is a certain risk of bias.

## 8 Conclusion

This study is the first bibliometric and visualization analysis of PFPS research over the past 23 years from multiple perspectives, providing a new perspective for a rapid understanding of the understanding of the PFPS field. The current state of research suggests that PFPS research still has a vast potential for growth. The most influential countries, institutions, journals, and authors are the USA, La Trobe Uni, Journal of Orthopaedic and Sports Physical Therapy, Rathleff MS and Barton CJ. The research hotspot keywords are hip-knee joint strength and core strength, lower limb kinematics and biomechanics, female (runners), muscle activation, risk factors, gait retraining, clinical practice guidelines, and rehabilitation. To a certain extent, these hot keywords reflect the trend of the field and the direction of cutting-edge research. However, there are still many opportunities and challenges in PFPS research, including novel exercise therapies (blood flow limiting training interventions, gait retraining interventions, and neuromuscular control training interventions) to improve the clinical efficacy of PFPS, gait retraining for prevention of PFPS in runners (especially women), and clinical efficacy of novel PRP formulations for the treatment of PFPS. Overall, this study presents a comprehensive and systematic overview of the extensive and in-depth literature resources related to PFPS in the form of a knowledge map. This helps scholars to conveniently gain a deeper understanding of the development and outlook of the field, which in turn promotes further research.

## Data availability statement

Publicly available datasets were analyzed in this study. This data can be found at: Web of Science.

## Author contributions

JX: Conceptualization, Formal analysis, Writing – original draft, Writing – review & editing. ZC: Methodology, Project administration, Writing – original draft. MC: Investigation, Resources, Software, Writing – review & editing. XW: Data curation, Supervision, Writing – review & editing. XL: Funding acquisition, Resources, Supervision, Writing – review & editing. YW: Formal analysis, Funding acquisition, Writing – review & editing.
